# Allicin and Omega-3 fatty acids attenuates acetaminophen mediated renal toxicity and modulates oxidative stress, and cell apoptosis in rats

**DOI:** 10.1007/s00210-023-02609-z

**Published:** 2023-07-12

**Authors:** Moamen Elsafty, Ahmed Abdeen, Mohamed Aboubakr

**Affiliations:** 1 Department of Pharmacology, Faculty of Veterinary Medicine, Benha University, Moshtohor, Toukh, 13736 Qaliobiya Egypt; 2 Department of Forensic Medicine and Toxicology, Faculty of Veterinary Medicine, Benha University, Moshtohor, Toukh, 13736 Qaliobiya Egypt

**Keywords:** Acetaminophen, Nephrotoxicity, Caspase-3, HSP70, Antioxidant, Allicin, Omega-3

## Abstract

Acetaminophen (APAP), a widely used medication known for its pain-relieving and fever-reducing effects, can cause kidney failure if taken in excess. To investigate the potential protective effects of allicin (ALC) and/or omega-3 fatty acids (O3FA) against acetaminophen-induced kidney damage, a study was conducted using 49 rats divided into seven groups. The control group was given saline, while the other groups received ALC, O3FA, APAP, ALC + APAP, O3FA + APAP, or ALC + O3FA + APAP. After administering APAP, the rats showed decreased levels of total protein and albumin in their blood, along with increased levels of creatinine and urea. The concentration of reduced glutathione (GSH), as well as the activity of superoxide dismutase (SOD) and catalase (CAT), decreased, while the level of malondialdehyde (MDA) in the renal tissues increased. The activation of caspase-3 and HSP70 also suggested an impact on kidney histopathology. Overall, the study found that ALC and/or O3FA may have a protective impact against acetaminophen-induced kidney damage through their anti-inflammatory, anti-apoptotic, and antioxidant defense systems.

## Introduction

Nephrotoxicity is a condition that arises when the kidneys are unable to effectively detoxify and excrete substances due to damage or destruction caused by toxic agents that come from outside the body (exogenous), are produced within the body (endogenous), or result from drug exposure (Kim and Moon 2012). Acetaminophen (N-acetyl-p-aminophenol) is one of the drugs that can cause nephrotoxicity, and it belongs to a class of medications known as antipyretics and analgesics (Baleni et al. 2015). Acetaminophen has a distinctive role among pain-relieving medications. In contrast to NSAIDs, it is widely accepted that acetaminophen does not possess any significant anti-inflammatory effects and is not associated with gastrointestinal damage (Bertolini et al. 2006). It is generally viewed as a safe medication, but excessive consumption of it can result in kidney damage, known as nephrotoxicity (Chinnappan et al. 2019).

When used in accordance with the recommended therapeutic doses, acetaminophen is generally considered to be free of adverse effects. However, prolonged use or exceeding the recommended dosage can result in liver and kidney damage, and in severe cases, it may even lead to death (Raoof et al. 2012). If consumed in large doses, APAP creates significant amounts of NAPQI, which can lead to increased production of ROS causing oxidative stress. This process occurs due to the depletion of the cellular antioxidant system, particularly glutathione (GSH) and GSH-dependent enzymes, which are overwhelmed by the excessive production of NAPQI (Abdel-Daim and Abdeen 2018; El-Maddawy and El-Sayed 2018). Both experimental animal models and humans commonly experience acute renal failure as a result of exposure to a toxic dosage of APAP (Dallak et al. 2022).

While hepatotoxicity is more commonly seen than nephrotoxicity in cases of APAP overdose, there is still a possibility of renal damage and acute failure of kidney even if the liver is not affected (Khan et al. 2021). While the precise mechanism of how APAP causes kidney damage is not fully understood, it is known that oxidative stress and the generation of ROS induced by NAPQI, markedly contribute to the development of renal injury caused by APAP (Ko et al. 2017). We postulated that in rats, the nephrotoxicity caused by APAP is likely associated with oxidative damage and lipid peroxidation caused by ROS. We further suggested that combining drug delivery with a potent antioxidant effect might be effective in reducing the harmful effects of APAP on the kidney.

Allicin (ALC) is the primary biologically active component of garlic and was the first compound isolated from it (Borlinghaus et al. 2014). Garlic contains various active constituents, with allicin (thio-2-propene-1-sulfinic acid S-allyl ester) being a major component. Allicin is derived from S-allyl cysteine-S-oxide (alliin), a stable precursor present in garlic, through the action of alliinase enzyme, which is activated when garlic cloves are crushed or macerated (Okada et al. 2006). Allicin has been demonstrated to exhibit antioxidant properties and to have various actions that may be beneficial for human health (El-Kashef et al. 2015; Sánchez-Gloria et al. 2021).

ALC's antioxidant activity is likely based on its capacity to eliminate oxygen free radicals (Ghanayem et al. 2005). ALC is a natural antioxidant that has the ability to scavenge hydroxyl and oxygen free radicals (Chung et al. 2013). Additionally, it can prevent lipid peroxidation induced by hydroxyl radicals in tissue homogenates. Moreover, ALC has been shown to inhibit the activity of cytochrome P450 enzyme CYP2E1, which is responsible for the formation of reactive toxic metabolites that can cause kidney damage in humans and animals (Das et al. 2010; Moore et al. 2013). We propose that allicin has the potential to mitigate the nephrotoxic effects and oxidative stress caused by acetaminophen.

Omega-3 polyunsaturated fatty acids (O3FA) are a type of compound that contains multiple double bonds. These O3FAs are considered essential in the diet and can be obtained from sources such as fish oil. Fish oil contains various O3FAs, such as alpha-linolenic acid (ALA), docosahexaenoic acid (DHA), and eicosapentaenoic acid (EPA) (Owumi et al. 2020). Omega-3 polyunsaturated fatty acids (O3FA) are present in various body components such as cell membranes and are involved in cell signaling, as well as exhibiting antioxidant and anti-inflammatory properties (Avramovic et al. 2012; de Batlle et al. 2012).

Multiple investigations have shown the potential of O3FA for mitigating oxidative stress and apoptosis, as well as their utility as pharmaceutical agents for managing inflammatory ailments (Li et al. 2017; Bäck and Hansson 2019; Lee and Kang 2019). The protective effect of O3FA on the kidneys has also been confirmed by studies (El-Ashmawy et al. 2018). Long-term use of O3FA has been shown in clinical studies to enhance renal function and decrease the likelihood of death or end-stage renal disease (Hassan and Gronert 2009).

The aim of this research is to explore whether ALC and/or O3FA can prevent kidney injury caused by APAP.

## Materials and methods

### Chemicals

Panadol^®^ was purchased from GlaxoSmithKline Pharmaceuticals Company located in Brentford, United Kingdom, and it contained 1 g of acetaminophen (APAP). Allicin was obtained in its pure powder form with a concentration of 35% from Delta Vet Center, located in Cairo, Egypt. Pure fish oil with a concentration of 100% Omega-3 fatty acids was obtained from Sigma Pharmaceutical Industries, also located in Cairo, Egypt. Bio-diagnostic Company in Giza, Egypt was the source of the purchased kits.

### Experimental animals

A total of 49 male Albino Wister rats weighing between 160–200 g and aged 2 months were obtained from the Center of Laboratory Animals, Faculty of Veterinary Medicine at Benha University, Egypt. Before experiment, the rats were given a period of 14 days to acclimatize themselves to the environment, which was maintained at a temperature of 25 °C. During this time, they were provided with a laboratory-standard commercial diet and allowed access to water ad libitum.

### Experimental design

The rats were divided into 7 groups, each consisting of 7 rats. The control group was given distilled water. The second group was orally given ALC at a dosage of 10 mg/kg BW, and the third group was given O3FA orally at a dosage of 100 mg/kg BW. The dosages of ALC and O3FA were determined from the studies carried out by (Abdel-Daim et al. 2019) and (Adeyemi and Olayaki 2018), respectively. The fourth group was given saline orally every day, and on the 27^th^ day of the experiment, a single oral dose of APAP at a dosage of 1 g/kg BW was administered to induce toxicity, as per the method outlined by (Abdeen et al. 2019) and designated as the APAP toxic control group. The fifth, sixth, and seventh groups were given ALC + APAP, O3FA + APAP, and ALC + O3FA + APAP, respectively. The rats in these groups were given ALC, O3FA, and APAP as described earlier. Saline, ALC, and O3FA were given for duration of 30 days.

### Sampling

After the completion of the experiment, the rats were anesthetized 24 h later, and blood samples were taken from the retro-bulbar venous plexus. Following this, all rats were euthanized, and their kidney tissues were removed and divided into two parts. One part was reserved for histopathological investigation, while the other part was preserved at a temperature of -80 °C for biochemical analysis within 1 week.

### Serum biochemical studies

The study measured various biochemical parameters, including creatinine, BUN, total protein, and albumin levels (Lowry et al. 1951; Coulombe and Favreau 1963; Larsen 1972; Young et al. 2001). The Bio-Diagnostic Company in Giza, Egypt, provided the kits used for these tests, and they were evaluated based on the manufacturer's instructions.

### Tissue homogenate preparation for oxidative markers evaluation

The tissue samples were washed using a solution of PBS (phosphate-buffered saline) that contained 0.16 mg/ml heparin in order to remove any red blood cells and clotting. To prepare the tissue samples for analysis, they were homogenized using a sonicator homogenizer with 5–10 ml buffer per gram of tissue, consisting of 50 mM potassium phosphate and 1 mM EDTA at pH 7.5. The supernatant obtained after centrifugation of the homogenates using a cooling centrifuge at 4000 rpm for 15 min was stored at a temperature of -80 °C. The study measured oxidative status by examining malondialdehyde (MDA) levels (Uchiyama and Mihara 1978), catalase (CAT) activity (Aebi 1984), superoxide dismutase (SOD) activity, and reduced-glutathione (GSH) levels (Beutler et al. 1963) using specialized diagnostic kits obtained from Biodiagnostic Company located in Egypt.

### Histopathological examinations

The right kidney tissue of each rat was immediately preserved in 10% neutral-buffered formalin for histopathological analysis. The kidneys were dehydrated, embedded in paraffin, and sliced into 5-μm sections. Hematoxylin and eosin (H&E) staining was used to examine the sections histologically, following the procedure explained by (Bancroft et al. 2013). The kidney tissue sections were finally observed using a light microscope from Leica, Germany.

### Immunological assays

The kidney tissue sections were heated in an oven at 60 °C for 25 min and then treated with xylene and graded alcohol to remove paraffin and rehydrate the tissue. The antigen retrieval process was carried out by boiling the sections in a 10 mM sodium citrate buffer in a microwave. The immunohistochemistry staining protocol was followed as per the manufacturer’s instructions (DakoCytomation, USA). First, endogenous peroxidase was blocked using 0.03% hydrogen peroxide sodium azide for 5 min. Then, the tissue sections were washed with a wash buffer and incubated overnight at 4 °C with polyclonal anti-HSP70 and anti-caspase 3 antibodies, applied at a dilution of 1:200 and 1:250, respectively. Afterward, the sections were incubated with avidin–biotin complex at 37 °C for 45 min, washed with a wash buffer and kept in a humid chamber. Streptavidin-HRP was then added, followed by diaminobenzidine-substrate chromagen and hematoxylin counterstaining. The sections were dipped in weak ammonia, washed, and cover slipped. The positively stained antigens appeared brown under light microscopy.

### Statistical analysis

The mean ± SD was used to present the data, and statistical analysis was performed using Graphpad Prism 9 software. This analysis involved one-way ANOVA, followed by Tukey's test. Statistical significance was determined when the probability value (*P*) ≤ 0.05.

## Results

### Biochemical study

**Fig. 1:**
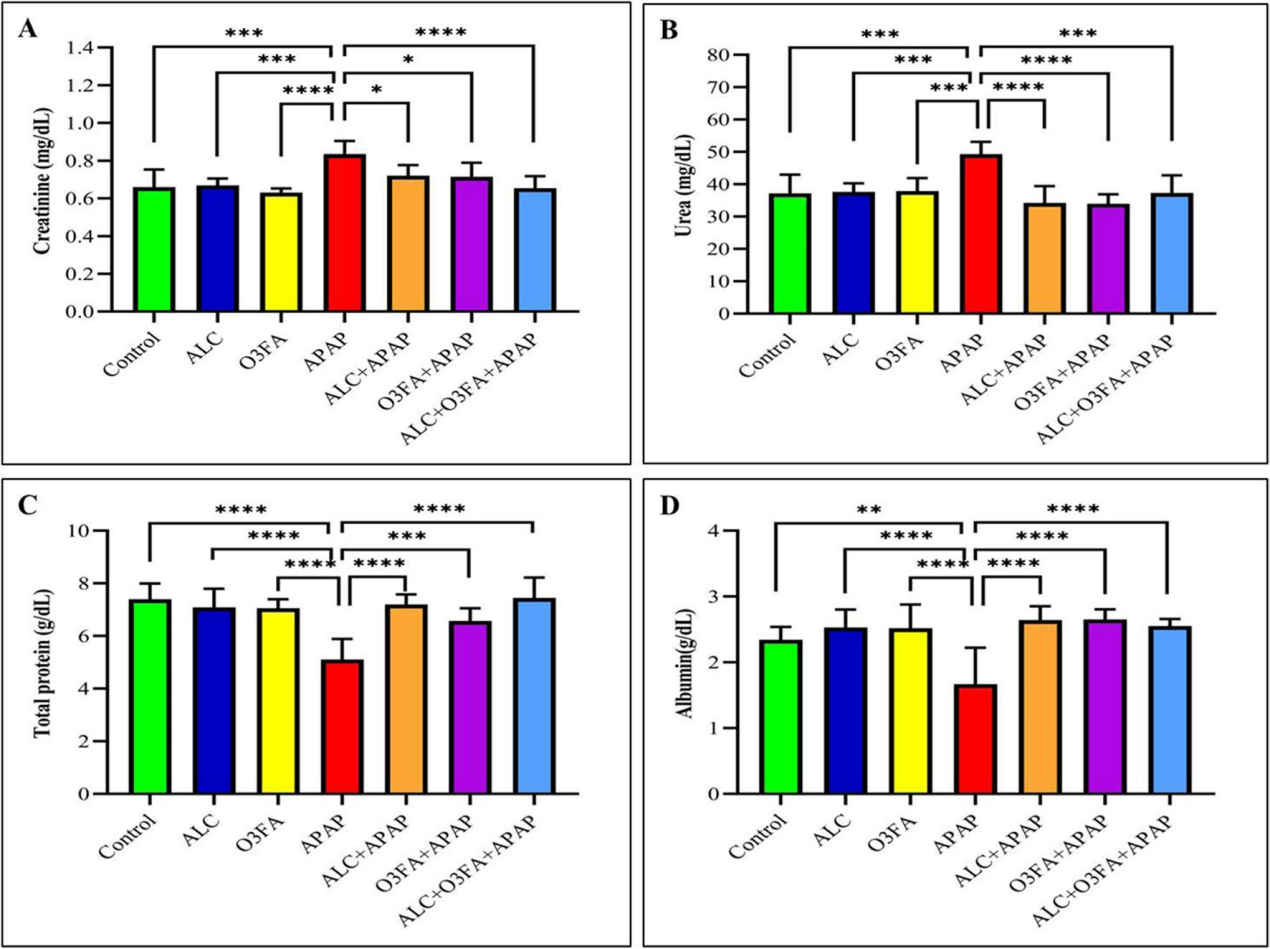
Effect of allicin (ALC), omega 3 fatty acid (O3FA), and acetaminophen (APAP) on renal biomarkers in the serum of rats (*n*=7). **A** (Creatinine); **B** (Urea); **C** (Total protein); **D** (Albumin). Stars indicated statistical differences between the APAP-treated group and other groups. * *P* ≤ 0.05; ** *P* ≤ 0.01; *** *P* ≤ 0.001; **** *P* ≤ 0.0001

Figure 1 indicates that the APAP-treated group had a significant rise in serum creatinine and urea levels, while experiencing a significant decrease in serum albumin and total protein levels compared to the control group. In contrast, the ALC + APAP, OMG-3 + APAP, and ALC + OMG-3 + APAP groups showed a significant reduction in urea and creatinine levels, while experiencing a significant increase in albumin and total protein levels compared to the APAP-treated group.

### Oxidative stress markers assay

**Fig. 2:**
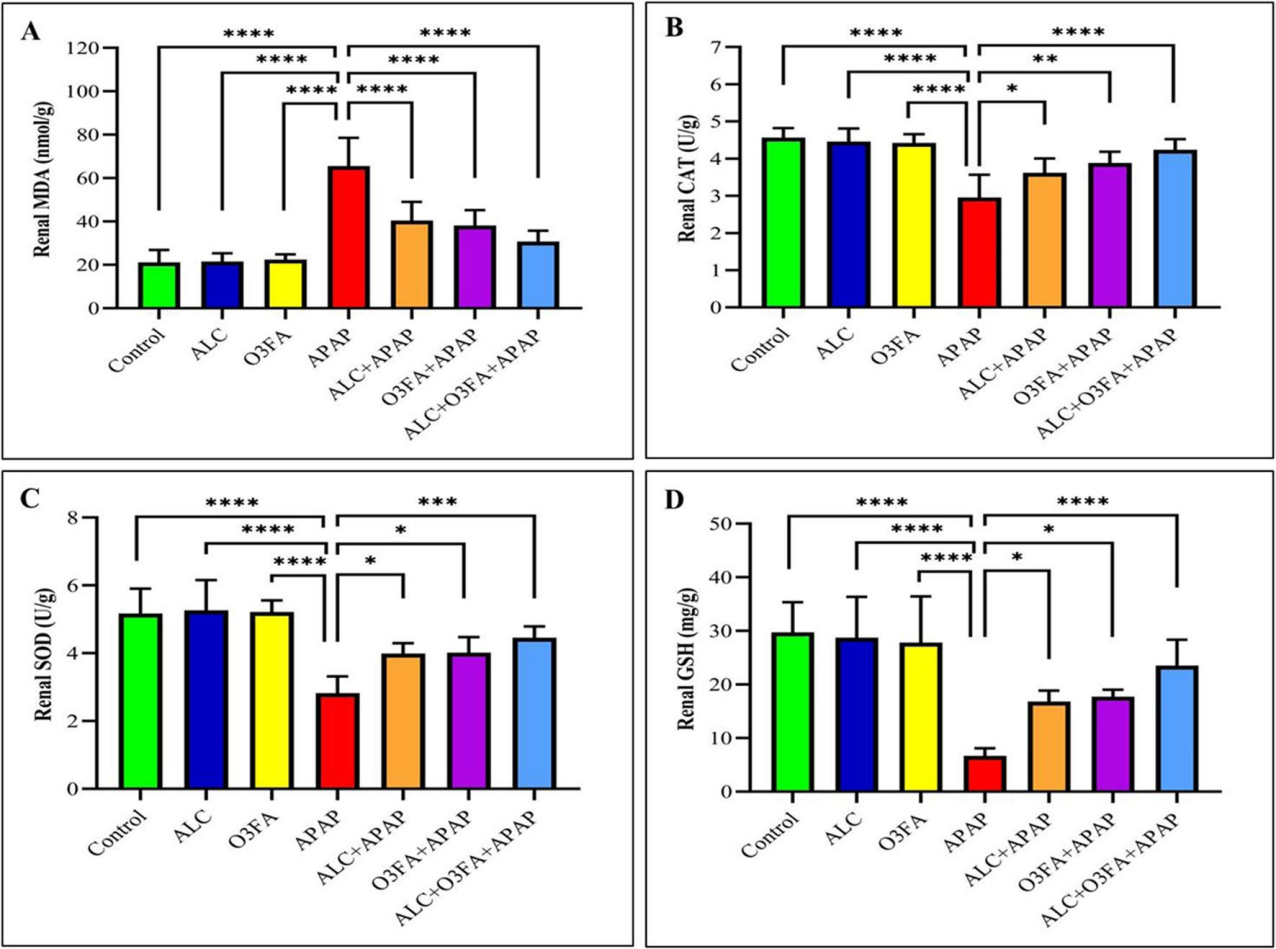
Effect of allicin (ALC), omega 3 fatty acid (O3FA), and acetaminophen (APAP) on renal antioxidants in renal tissues of rats (*n*=7). **A** (MDA); **B** (CAT); **C** (SOD); **D** (GSH). Stars indicated statistical differences between the APAP-treated group and other groups. * *P* ≤ 0.05; ** *P* ≤ 0.01; *** *P* ≤ 0.001; **** *P* ≤ 0.0001

Figure 2 depicts the influence of APAP-induced toxicity and treatment with allicin and/or omega-3 on oxidative parameters and lipid peroxidation in the kidney. The findings indicate that there was a significant increase in MDA levels, as well as a decrease in CAT, SOD, and GSH levels in the renal tissues of APAP-intoxicated rats in comparison to control rats. Nonetheless, the administration of ALC and/or O3FA effectively restored the adverse effects of APAP on renal MDA, CAT, SOD, and GSH levels to nearly normal levels.

### Histopathological changes of kidney

**Fig. 3:**
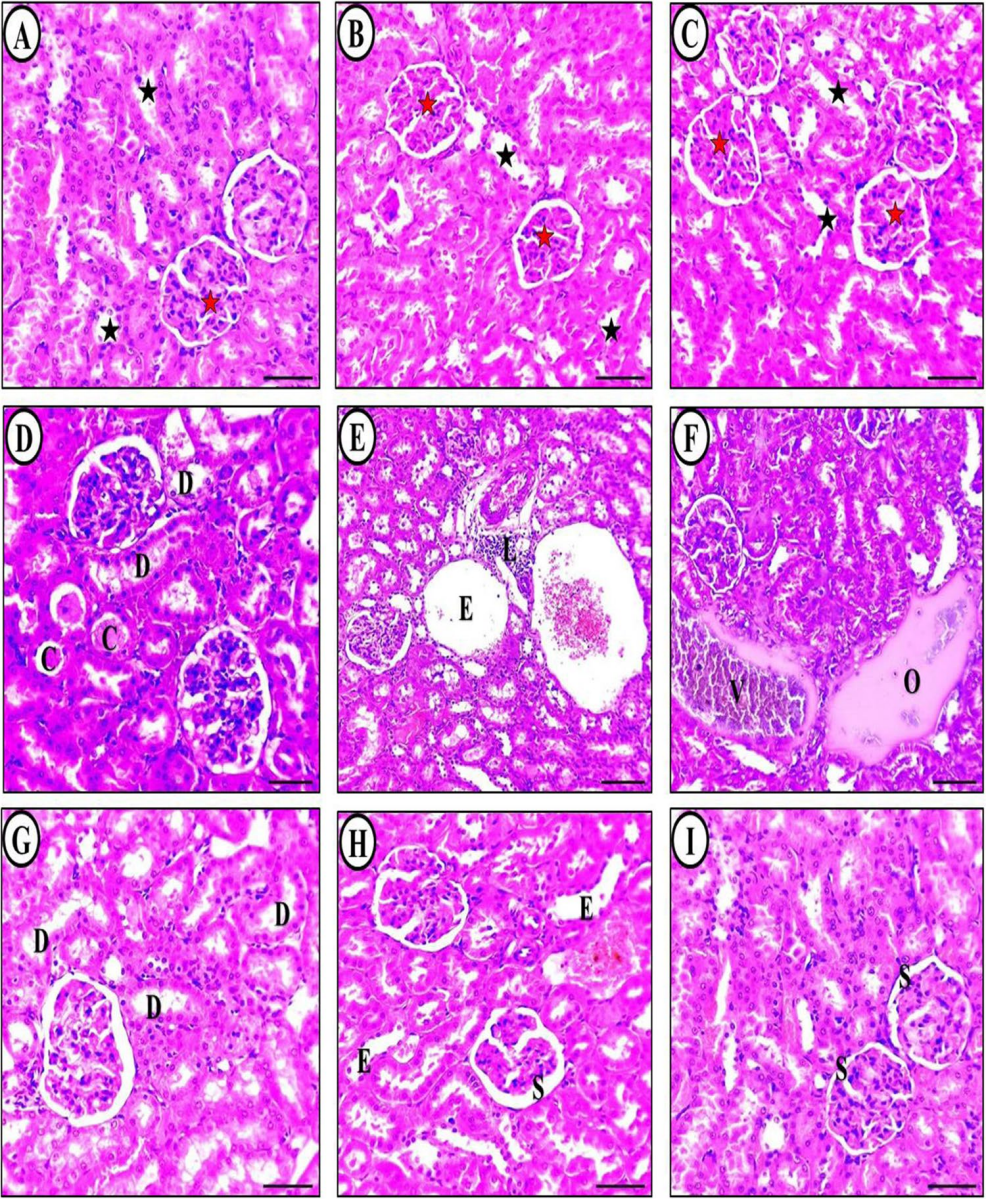
Light microscopic micrographs of rat kidney sections stained with H&E obtained at the end of the experiment from control (**A**), ALC (**B**), and O3FA (**C**) groups. Structure of kidney glomerular (red star), and renal tubules (black star) with normal histological structure and intact well-organized cellular boundary. Acetaminophen control positive group showing severe loss of brush border, tubular casts (C), tubular degeneration (D) (**D**), tubular cystic enlargement and lymphocytic infiltration (L) (**E**). Congested renal blood vessels (V) with proteinaceous fluid deposition (O) (**F**) were also seen. ALC + APAP treated group showed slightly to mild degenerations (D) (**G**). O3FA + APAP treated group showed slightly to mild constriction of renal corpuscles (S) and tubular enlargement (E) (**H**). ALC + O3FA + APAP treated group had a better morphology with less tubular necrosis (**I**). The structures of kidney were comparable to the control group

At the end of the experiment, kidney tissues were collected from each group of animals and subjected to H&E staining, followed by examination under a light microscope. The kidneys of the control group (Fig. 3A) showed normal proximal and distal convoluted tubules of renal corpuscles. The microscopic morphology of the kidneys in the allicin (Fig. 3B) and omega-3 (Fig. 3C) groups were similar to the control group. However, in the acetaminophen-treated group, there was a significant increase in glomerulus space widening, tubular dilatation, cellular debris, vacuolization, and extensive tubular epithelial degeneration. Many tubules had hyaline casts, desquamated cells, and necrotic cell debris. Additionally, the interstitium of the renal cortex showed congested blood vessels and extravasation (Fig. 3D-F).

The ALC + APAP group (Fig. 3G) and OMG-3 + APAP group (Fig. 3H) showed a better morphology with less tubular necrosis than the untreated positive control group. The renal corpuscles and convoluted tubules had a normal structure, with some mildly dilated glomeruli spaces in a few fields. There was also a restoration of the brush border at the apex and narrow lumina in many tubules, and the presence of mild tubular epithelial vacuolization, luminal cast formation, and cell desquamation was hardly detected. The ALC + O3FA + APAP treatment group (Fig. 3I) showed preserved normal structure of renal corpuscles and convoluted tubules with a few mildly dilated glomeruli spaces in some fields.

**Fig. 4:**
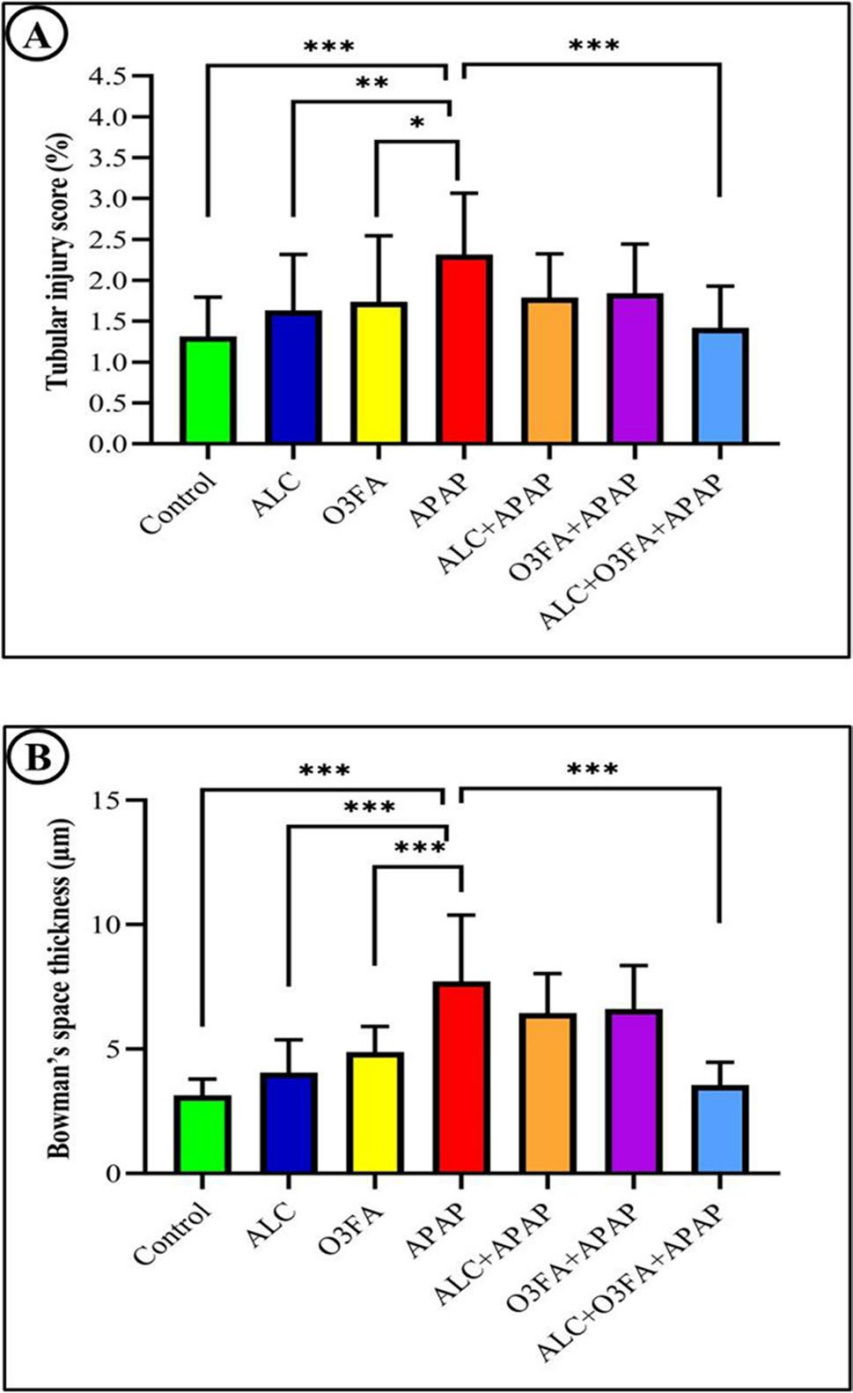
Bar graph of tubular injury score (**A**). Tubular damage and necrosis was significantly (*P < 0.05) reduced in kidneys of rats treated with ALC + O3FA + APAP. Data are expressed as mean ± SD for each treatment group. Quantitative analysis of the mean thickness of Glomeruli’s space (**B**)

A pathologist evaluated the percentage of tubular injury using a 4-point scale based on epithelial flattening, tubular dilatation, and brush border loss observed in ten randomly selected, non-overlapping fields. The scoring system assessed only cortical tubules and graded the degree of injury from 0 to 5, with 0 indicating no injury and 5 indicating severe injury (greater than 75% of tubular injury). 0 = no tubular injury; 1 = 10% tubular injury; 2 = 10%-25% tubular injury; 3 = 26%-50% tubular injury; 4 = 51%-75% tubular injury; and 5 =  > 75% tubular injury (Fig. 4A). The mean thickness of the glomerular space in the ALC + OMG-3 + APAP group was also measured using Image J 1.53q image analyzer in 10 non-overlapping high-power fields/rat of H&E-stained sections, and it was significantly reduced compared to the control group, although it was still significant (Fig. 4B).

#### Immunohistochemical analysis (caspase-3 expression)

**Fig. 5:**
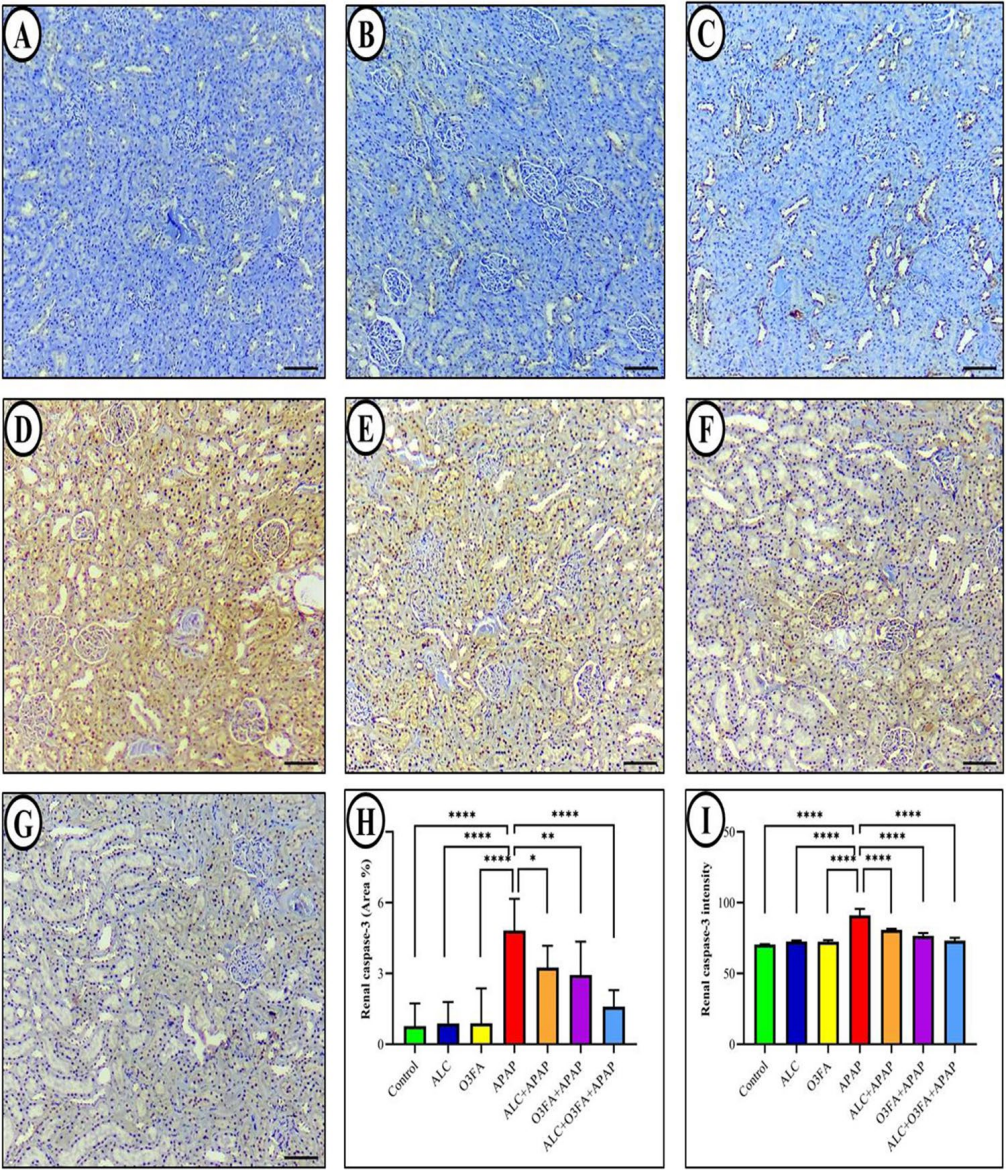
Immunohistochemical staining of caspase-3 in rat kidney from: Control group (**A**), ALC alone group (**B**), O3FA alone group (**C**), APAP-intoxicated group (**D**), ALC + APAP group (**E**), O3FA + APAP group (**F**), ALC + O3FA + APAP group (**G**). Immunostaining was performed using anti-caspase-3 antibody and developed with DAB. Brown color indicates caspase-3 positivity. Bar graph of caspase-3 immunohistochemical expression in the different study groups; area percent of immunoreactivity of caspase-3 (**H**), and renal caspase-3 intensity (**I**)

Immunohistochemical analysis was performed to examine the expression of caspase-3 in the renal epithelium of different rat groups. In the kidney sections of normal control rats, there was very little caspase-3 immune-reactivity observed. However, in the APAP-intoxicated group, there was a significant increase in caspase-3 expressions when compared to the control group. Semi-quantitative analysis showed that the co-administration of ALC and O3FA significantly reduced caspase-3 expressions compared to the APAP-intoxicated group. These changes in caspase-3 protein expression were consistent with the pathological damage observed in the rats' kidneys, as shown in Fig. 5.

#### Immunohistochemical analysis (HSP70 expression)

**Fig. 6:**
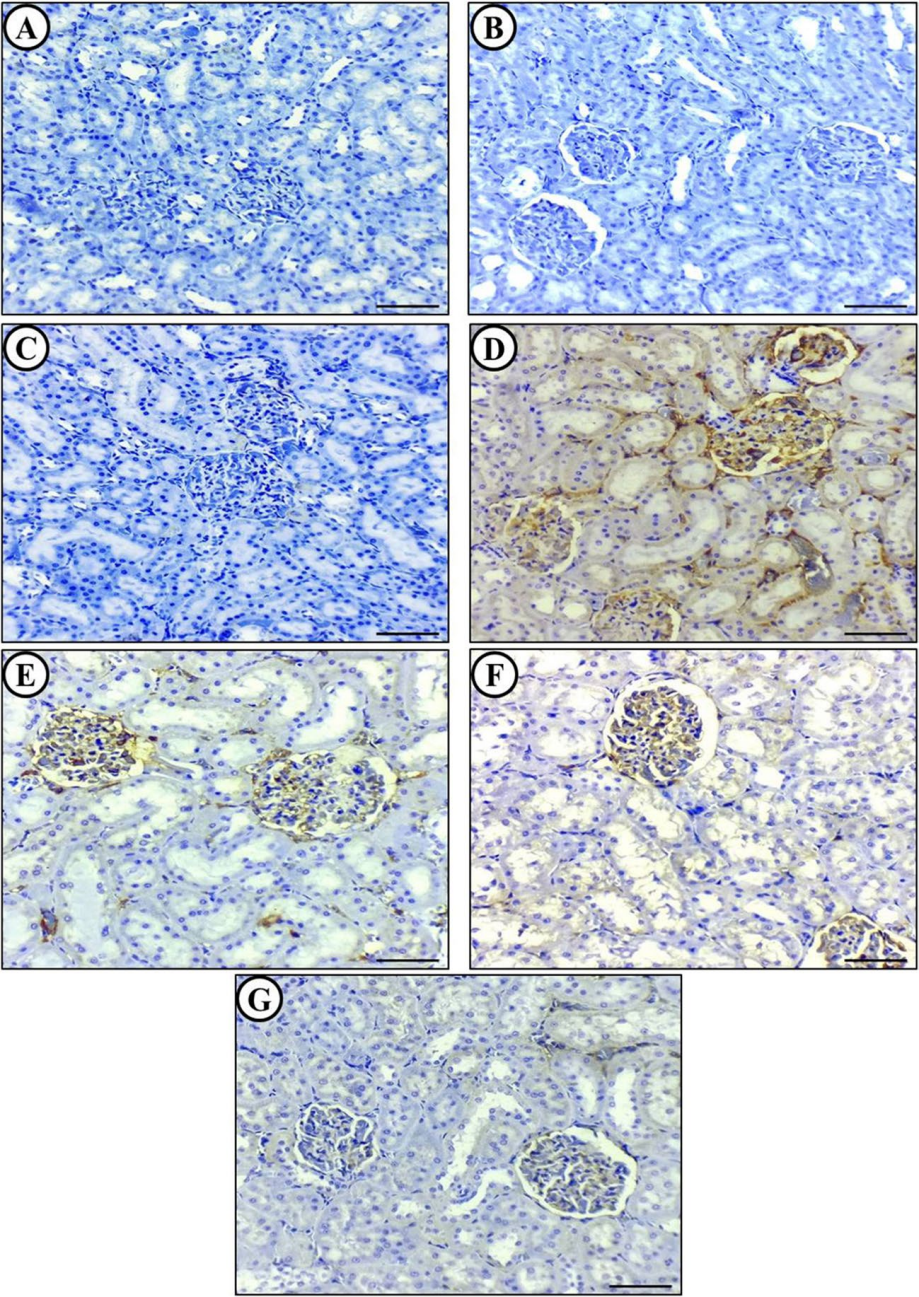
Cross section in the kidney of APAP induced nephrotoxicity model showing changes in renal HSP70 Immunohistochemical expression after treatment with ALC and/or O3FA. Control group (**A**), ALC alone group (**B**), O3FA alone group (**C**); all showing no expression of HSP70 in the cortical regions of kidney. APAP-intoxicated group showing diffuse intense expression (**D**), ALC + APAP group (**E**), O3FA + APAP group (**F**); both showing moderate immunopositivity, and ALC + O3FA + APAP group showing partial inhibition of caspase-3 expression as evidenced by weak immune staining in the cortical regions (**G**). Brown color indicates immunopositivity

The immunohistochemical analysis indicated that the renal tissue of rats in the APAP-intoxicated group showed a significant increase in HSP70 protein expression compared to the control group where there was no expression of HSP70 protein in rats' kidney. The HSP70 proteins were observed to spread from the kidney cells and were disseminated in the kidney tubules and renal interstitium. The kidney sections of rats treated with ALC or O3FA alone showed partial inhibition of HSP70 expression, which was indicated by weak immune staining in the cortical regions. Additionally, the kidney sections from the group that received co-treatment with ALC + O3FA + APAP showed the least or modest expression of HSP70 protein in rats' kidney as shown in (Fig. 6).

## Discussion

The kidney is an essential organ that has a vital function in eliminating waste products, regulating fluid volume, and balancing electrolytes. Given that APAP is commonly used as a painkiller and fever reducer, it's important to evaluate its potential toxicity. Previous studies have shown that APAP can cause acute kidney injury in both humans (Mour et al. 2005) and animal models (Karaali et al. 2018).

Although APAP is generally considered safe when used in therapeutic doses, its overdose is a leading cause of liver damage and death due to drug toxicity in many parts of the world (Yayla et al. 2014). The harmful effects of APAP overdose on the liver and kidneys are the result of a complex series of events (Hinson et al. 2010). In most cases, APAP-induced kidney damage becomes evident after liver damage, but it is important not to overlook the possibility of renal tubular injury and acute renal failure occurring independently of liver injury (Eguia and Materson 1997).

Our research had two main objectives: firstly, to examine whether an excessive amount of acetaminophen (APAP) can lead to alterations in the structure of the glomerulus; and secondly, to determine whether the combination of two antioxidants, ALC and O3FA, can provide protection against APAP-induced alterations to the ultrastructure and an increase in biomarkers of acute kidney damage. It is widely recognized that the consumption of high doses of APAP can be detrimental to kidney function due to the buildup of a toxic metabolite called NAPQI. The toxicity induced by NAPQI in the kidneys is a result of oxidative stress, which occurs due to an increase in reactive oxygen species (ROS), leading to the oxidation of cellular macromolecules such as lipids, proteins, and DNA, as well as mitochondrial dysfunction (Murad et al. 2016; El-Maddawy and El-Sayed 2018; Sohail et al. 2022).

Urea and creatinine are important indicators of kidney damage in clinical practice (Refaie et al. 2014; Uthra et al. 2017) and are commonly used as sensitive markers to diagnose kidney diseases. In our study, we measured serum urea and creatinine levels to assess kidney damage. The results showed that APAP intoxication in rats significantly increased the levels of urea and creatinine compared to the control group. Additionally, levels of albumin and total protein were significantly reduced in the APAP-intoxicated group compared to the control group, indicating that kidney function had deteriorated. These findings are consistent with previous studies that have reported increased serum urea and creatinine levels as markers of APAP-induced kidney damage (Karthivashan et al. 2016; Abdeen et al. 2019; Sohail et al. 2022).

The body's antioxidant capacity, which helps protect against damage caused by oxidative stress, is often measured by biomarkers such as SOD, GSH, CAT, and MDA. Oxidative stress is typically characterized by increased levels of lipid peroxidation and changes in the non-enzymatic and enzymatic antioxidant systems. In our study, we evaluated changes in tissue levels of MDA, GSH, CAT, and SOD activity as indicators of the oxidative stress caused by oral administration of acetaminophen. The results showed an increase in lipid peroxidation, as well as a significant decrease in GSH levels. Additionally, reductions in CAT levels and SOD activity were observed.

GSH, a tripeptide found in many mammalian tissues, plays a critical role in scavenging free radicals and NAPQI, which is a reactive intermediate of acetaminophen. It is a crucial component of the body's antioxidant defense system and eliminates harmful free radicals like hydrogen peroxide and superoxide radicals while protecting membrane protein thiols (Yayla et al. 2014). Our study showed that APAP overdose led to an increase in renal MDA levels and a significant inhibition of major renal antioxidant enzymes (GSH, SOD, and CAT) activities. The decrease in SOD, CAT, and GSH levels, along with an increase in MDA levels, is consistent with previous research on APAP-induced nephrotoxicity (Canayakin et al. 2016; Abdeen et al. 2019; Eshrati et al. 2021; Wans et al. 2021).

Allicin, derived from garlic, is an organic disulfide compound that is responsible for its biological activity (Borlinghaus et al. 2014). According to a study by (Maldonado et al. 2003), a derivative of allicin called allyl cysteine was found to have protective effects against acute renal failure induced by gentamicin in rats by preserving antioxidant enzymes in the renal cortex. Other research has shown that ALC has hepatorenal protective effects due to its antioxidant properties, ability to scavenge ROS, immunomodulatory effects, and anti-inflammatory activities (Mehmetçik et al. 2008; El-Kashef et al. 2015).

The study results show that rats given ALC had lower levels of serum urea and creatinine, and higher levels of albumin and total protein, compared to the APAP-intoxicated group. This suggests that ALC provided some protection against APAP-induced kidney toxicity and helped to bring these markers closer to normal levels.

Allicin has been reported to have a protective effect against oxidative stress by scavenging free radicals and reducing cytotoxic compounds (Chan et al. 2013). In the current study, it was found that allicin could alleviate APAP-induced oxidative stress in renal tissue by reducing lipid peroxidation and preserving antioxidant biomarkers. Specifically, the addition of ALC led to a significant decrease in MDA levels, which is an end product of lipid peroxidation, compared to the APAP group.

The current study's results align with those of (Şener et al. 2000), who reported that an aqueous garlic extract was able to prevent the increase in MDA levels and restore them to normal levels. GSH is a critical component of intracellular protective mechanisms against oxidative stress and other harmful stimuli (Moskovitz et al. 2002). SOD is an enzyme that contains metal ions and plays a critical role in neutralizing superoxide anions by converting them into oxygen and hydrogen peroxide. This mechanism is considered one of the most efficient antioxidant defense systems, and SOD is the first enzyme involved in this process (Salvemini et al. 2002). The administration of ALC increased the levels of GSH, SOD, and CAT compared to those in the APAP-intoxicated group. These results suggest that the protective effects of ALC may be due to its antioxidant and anti-apoptotic properties.

Over the past thirty years, there has been a significant increase in scientific understanding of O3FA. Recent research indicates that fish oil containing high levels of O3FA can help slow the progression of various diseases such as cancer, depression, arthritis, asthma, as well as cardiovascular and kidney disorders (De Caterina et al. 1994). Additionally, the protective effect of O3FA on kidneys has been well-documented (El-Ashmawy et al. 2018), with clinical studies suggesting that long-term O3FA treatment can improve kidney function and reduce the risk of death or end-stage renal disease (Hassan and Gronert 2009).

The group treated with APAP showed a marked increase in serum creatinine and urea levels, as well as a significant decrease in serum albumin and total protein levels, indicating renal damage. However, treatment with O3FA improved renal function and had renoprotective effects by normalizing these biomarker levels. The renal damage caused by APAP toxicity is primarily associated with the overproduction of ROS and a reduction in antioxidant capacity, as indicated by several studies (Hasanein and Sharifi 2017; Eraky and El-Magd 2020; Eshrati et al. 2021). Allicin treatment also resulted in a significant reduction in serum creatinine and urea levels in cisplatin-intoxicated rats, according (Abdel-Daim et al. 2019).

The increase in ROS causes damage to cell membranes through lipid peroxidation, which results in the breakdown of polyunsaturated fatty acids (El-Ashmawy et al. 2018). This damage was observed in the current study by the increase in renal MDA concentration, which is an indicator of lipid peroxidation, and the reduction in renal GSH, SOD and CAT levels. The renoprotective effects of O3FA were shown by its ability to decrease MDA concentration and suppress lipid peroxidation while increasing renal GSH, SOD and CAT levels. Previous research has also shown that O3FA has antioxidant potential (Ali and Rifaai 2019). Similarly, allicin was found to have significant nephroprotective effects against cisplatin intoxication, which may be due to its antioxidant and anti-inflammatory activities (Abdel-Daim et al. 2019). The antioxidant potential of O3FA observed in this study is consistent with previous research findings (Emam et al. 2018; Amos et al. 2019; Owumi et al. 2020).

The use of ALC and/or O3FA resulted in significant improvement in the histopathological changes caused by APAP. However, the tubular scores of the ALC and/or O3FA group were still significantly higher than those of the control group, indicating that there was still some damage present despite the ameliorative effects of the treatments. Additionally, it is noteworthy that the effects of ALC and/or O3FA were very similar.

Our biochemical findings and indications of oxidative stress were confirmed by histological examinations in this study. The acute overdose of APAP resulted in evident nephrotoxicity, which was demonstrated through acute tubular necrosis and severe kidney damage, such as congestion, degeneration, and loss of brush border. These changes in the kidney structure may have been responsible for the lipid peroxidation induced by APAP. These results are consistent with previous studies that described renal histological changes after APAP administration (El-Maddawy and El-Sayed 2018; Reshi et al. 2020; Eshrati et al. 2021; Wans et al. 2021; Dallak et al. 2022; Sohail et al. 2022). However, treatment with ALC and/or O3FA preserved the normal structure of renal tubules, with only a few mildly dilated glomeruli spaces in some fields. The results suggest that the administration of ALC and/or O3FA effectively mitigated the renal damage induced by acetaminophen toxicity.

The increase in caspase-3 detected through immunohistochemistry in rats intoxicated with APAP suggests the presence of apoptosis. Studies have shown that APAP can cause the loss of mitochondrial membrane potential, which releases cytochrome c and triggers caspase-3 activation, leading to renal cell death (Das et al. 2010). Similar results when examining caspase-3 expression in APAP-induced renal injury in rats (Abdeen et al. 2019). Their study showed that the proximal tubules in the kidney were more severely affected than other parts of the kidney, which is consistent with our findings. This may be due to the high energy demand required for active transport in the proximal tubules, which are rich in mitochondria. The hypothesis is that APAP-induced oxidative damage and apoptotic mechanisms primarily target mitochondria because these organelles are the main site of ROS production and the initiation of apoptosis (Galvan et al. 2017).

There have been several research studies indicating the potential use of O3FA as a treatment for inflammatory diseases and for alleviating oxidative stress and apoptosis. Examples of such studies include those conducted by (Li et al. 2017; Lee and Kang 2019; Owumi et al. 2020). Nevertheless, administering ALC and/or O3FA prior to APAP exposure lowered caspase-3 expression, suggesting that these substances possess anti-apoptotic effects against APAP-induced nephrotoxicity by impeding mitochondria-mediated apoptosis. Orabi et al. (2020) also reported similar findings where the expression of activated caspase-3 protein was significantly reduced in renal tissues of rats treated with both diclofenac and allicin.

The immunohistochemical analysis of HSP70 expression revealed that the APAP-intoxicated group of rats had a significant increase in HSP70 protein expression in their renal tissue compared to the control group, which had no expression of HSP70 protein in their kidneys. Some HSP70 proteins were observed to spread from kidney cells and were present in kidney tubules and renal interstitium. In contrast, the kidney sections of the ALC-only or O3FA-only treated groups showed a partial inhibition of HSP70 expression with weak immune staining in the cortical regions. Furthermore, the rats' kidney sections from the co-treated group with ALC and O3FA had the lowest or modest expression of HSP70 protein.

The combination of ALC and O3FA had a synergistic effect in reducing oxidative stress and inflammation, as well as inhibiting caspase-3 and HSP70 expression, which was more effective than either treatment alone. These results suggest that both ALC and O3FA have potential as nephroprotective agents, and their combination could provide enhanced cytoprotection. Further studies on the chemical and biological interactions between these two agents are recommended.

## Conclusion

The current research revealed that an overdose of APAP induced renal damage, as evidenced by increased levels of kidney function tests and oxidative stress markers, elevated expression of apoptotic proteins, and observed histopathological changes in the renal tissue. However, ALC and/or O3FA were found to have a protective effect against APAP-induced renal damage by reversing all the biochemical and histopathological changes induced by APAP. This protective effect is mainly due to the antioxidant and anti-apoptotic activities of ALC and/or O3FA.

## Data Availability

The data presented in this study are available upon request from the first author.
